# Takotsubo cardiomyopathy recurrence with left ventricular apical ballooning following isolated right ventricular involvement: A case report

**DOI:** 10.3892/etm.2013.1112

**Published:** 2013-05-14

**Authors:** BYUNG-HYUN JOE, HUI-JEONG HWANG, CHANG-BUM PARK, EUN-SUN JIN, IL-SUK SOHN, JIN-MAN CHO, CHONG-JIN KIM

**Affiliations:** Cardiovascular Center, Kyung Hee University Hospital at Gangdong, Seoul 134-727, Republic of Korea

**Keywords:** Takotsubo cardiomyopathy, apical ballooning syndrome, right ventricle

## Abstract

We report a case of Takotsubo cardiomyopathy, which involved the right ventricle at first presentation and demonstrated involvement of the left ventricle during recurrence. The patient was admitted to Kyung Hee University Hospital due to a left hip fracture, which was considered a result of physical stress. Complete recovery was confirmed by echocardiography prior to recurrence. The cause of the second event was surgery for the left hip fracture. Recurrence of Takotsubo cardiomyopathy at various cardiac locations provides evidence against the existing hypotheses that variants of Takotsubo cardiomyopathy are associated with anatomically different distributions of cardiac adrenergic receptors, the degree of stimulation by sympathetic activity and different susceptibilities to such sympathetic stimulation.

## Introduction

Takotsubo cardiomyopathy is a transient ventricular akinesia without the appearance of coronary artery disease of sufficient magnitude to explain the extent of cardiac dysfunction (1). Typically, it occurs on the left ventricular (LV) apex and normally presents as apical ballooning with hypercontraction of basement segments. It is often accompanied by chest pain, dynamic reversible electrocardiographic abnormalities and mild elevation of cardiac enzyme levels (2). However, variants of Takotsubo cardiomyopathy have been described (3,4). We present the case of an 83-year-old woman who demonstrated isolated right ventricular (RV) involvement of Takotsubo cardiomyopathy at the first presentation and LV involvement during recurrence.

## Case report

**Figure 1.**
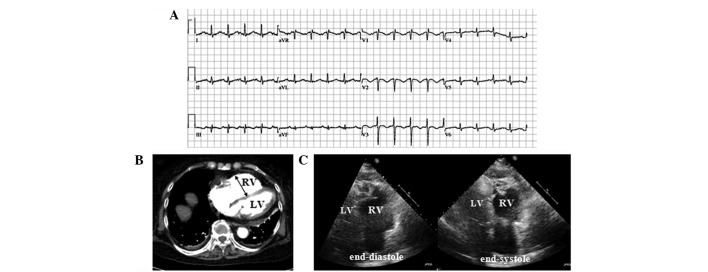
Right ventricular involvement of Takotsubo cardiomyopathy at first presentation. (A) Electrocardiogram, (B multi-slice computed tomography and (C) echocardiographic images. LV, left ventricle; RV, right ventricle.

**Figure 2.**
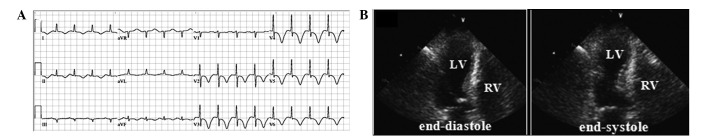
Left ventricular involvement of Takotsubo cardiomyopathy during recurrence. (A) Electocardiogram and (B) echocardiographic images. LV, left ventricle; RV, right ventricle.

An 83-year-old female patient with a history of hypertension and diabetes mellitus presented to the emergency department at Kyung Hee University Hospital (Seoul, Korea) with a left hip fracture resulting from a fall. The patient complained of chest discomfort and mild dyspnea. On admission, the patient’s blood pressure was 128/83 mmHg and heart rate was 108 beats/min. Electrocardiography (ECG) revealed sinus tachycardia with an RSR pattern in lead V1, T wave inversion and poor R progression in leads V1-3 and S1/Q3 (Fig. 1A). Serum troponin I was elevated to 0.380 ng/ml. Multi-slice computed tomography pulmonary angiography demonstrated mild dilatation of the right atrium (RA) and right ventricle, as well as mild pulmonary edema with small bilateral pleural effusions (Fig. 1B); however, there was no filling defect in the pulmonary arteries. Transthoracic echocardiography (TTE) revealed akinesia of the apico-mid RV free wall with RV dilatation (Fig. 1C) and a mild reduction of RV systolic function. LV ejection fraction was preserved (65%) without regional wall motion abnormality. The patient’s coronary angiography was normal. Potential malignancy was excluded by chest and abdominal computed tomography. The symptoms of chest discomfort and mild dyspnea resolved following conservative treatment. Follow-up TTE on the sixth day of hospitalization revealed normalized RV size and function, as well as resolution of the RV wall motion abnormality. The patient underwent surgery for the left hip fracture. On the day after surgery, the patient complained of chest pain and dyspnea. The patient’s blood pressure, heart rate and respiratory rate were 91/59 mmHg, 104 beats/min and 25 breaths/min, respectively. On ECG, deep T wave inversion was observed to be newly developed in leads I, aVL and V2-6 (Fig. 2A). Troponin I levels were elevated to 0.226 ng/ml. Portable echocardiography revealed akinetic and dilated LV apex and hyperkinetic basal segments with an LV ejection fraction of 56% (Fig. 2B). The RV function and wall motion was normal. Following conservative treatment, follow-up TTE revealed a good LV systolic function with normal wall motion. The patient was discharged uneventfully. The study was approved by the IRB ethics committee (KHNMC IRB 2013-027)

## Discussion

By considering the clinical course and the actual findings, the patient was diagnosed with recurrent Takotsubo cardiomyopathy, which involved the right ventricle at initial presentation and the left ventricle during recurrence. The provoking factor was considered to be an acute medical illness or intense physical stress.

The pathophysiology of Takotsubo cardiomyopathy has not been clearly established. A number of studies have suggested that an induced severe transient mid-ventricular cavity dynamic gradient and catecholamine-induced reduction in subendocardial blood flow lead to significant LV outflow tract obstruction and secondary ischemia of the LV apex and anterior wall in Takotsubo cardiomyopathy (5,6). However, this finding may be a phenomenon observed in only certain patients with LV involvement of Takotsubo cardiomyopathy (7,8). It does not explain the development of other variants of Takotsubo cardiomyopathy. Other studies have suggested that an anatomically different distribution of cardiac adrenergic receptors, the degree of stimulation by sympathetic activity and different susceptibilities to sympathetic stimulation are responsible for the development of variants (9,10). However, the present observation of a patient with Takotsubo cardiomyopathy involving different cardiac locations during recurrence may provide evidence against this suggestion.

Elesber *et al* (11) reported that patients with a clinical manifestation of biventricular involvement of Takotsubo cardiomyopathy differ from patients with LV involvement, which is associated with lower LV ejection fraction, longer hospitalization and more complications, including severe congestive heart failure, intra-aortic balloon pump and cardio-pulmonary resuscitation. Additionally, isolated RV Takotsubo cardiomyopathy may represent a distinct manifestation compared with LV Takotsubo cardiomyopathy, including acute right heart failure (3). Unfortunately, cases with isolated RV involvement are rarely reported (3,12). Therefore, further verification is required by additional observations in the future.

The prognosis of Takotsubo cardiomyopathy is considered favorable. However, it may be fatal and recurrent Takotsubo cardiomyopathy may cause several problems, including the misdiagnosis of acute coronary syndrome or sepsis, leading to incorrect management, repetitive symptoms, interruption of treatment for original medical problem and longer periods of hospitalization.

In conclusion, we report a unique case demonstrating recurrence with LV apical involvement following the occurrence of isolated RV Takotsubo cardiomyopathy. To our knowledge, this is the first report to describe such a case. The pathophysiology may be different from previous suggested mechanisms of Takotsubo cardiomyopathy.
